# Check the Head: Emergency Ultrasound Diagnosis of Fetal Anencephaly

**DOI:** 10.5811/westjem.2016.5.30326

**Published:** 2016-07-05

**Authors:** John W. Hall, Nicolas Denne, Joseph J. Minardi, Debra Williams, BJ Balcik

**Affiliations:** West Virginia University School of Medicine, Department of Emergency Medicine, Morgantown, West Virginia

## Abstract

**Background:**

Early pregnancy complaints in emergency medicine are common. Emergency physicians (EP) increasingly employ ultrasound (US) in the evaluation of these complaints. As a result, it is likely that rare and important diagnoses will be encountered. We report a case of fetal anencephaly diagnosed by bedside emergency US in a patient presenting with first-trimester vaginal bleeding.

**Case Report:**

A 33-year-old patient at 10 weeks gestation presented with vaginal bleeding. After initial history and physical examination, a bedside US was performed. The EP noted the abnormal appearance of the fetal cranium and anencephaly was suspected. This finding was confirmed by a consultative high-resolution fetal US. Making the diagnosis at the point of care allowed earlier detection and more comprehensive maternal counseling about pregnancy options. This particular patient underwent elective abortion which was able to be performed at an earlier gestation, thus decreasing maternal risk. If this diagnosis would not have been recognized by the EP at the point of care, it may not have been diagnosed until the second trimester, and lower-risk maternal options would not have been available.

## INTRODUCTION

Vaginal bleeding during pregnancy is a common complaint encountered by emergency physicians (EP) and one that can be anxiety inducing for patients and their families. Approximately 20%–40% of pregnant women will experience some amount of vaginal bleeding during their first 20 weeks.^1^ Approximately 1.6% of all emergency department visits can be attributed to vaginal bleeding during early pregnancy. Most commonly, vaginal bleeding during early pregnancy can be attributed to ectopic pregnancy, threatened/complete/incomplete abortion, physiologic implantation of the pregnancy, or some uterine/cervical structural abnormality. EPs have been shown to be capable of accurately determining the presence of an intrauterine pregnancy using ultrasound (US), and ultrasound is commonly employed by EPs.

Early pregnancy complaints are common and can create a diagnostic challenge for the EP. EPs are more commonly using point-of-care ultrasound (POCUS) to evaluate these and a multitude of other complaints. US has been shown to detect common etiologies of first-trimester bleeding;^3^ however, there are some uncommon and important diagnoses that, if recognized early, may have important implications in patient care. With the increasing use of US by EPs, it is likely these uncommon but important diagnoses will be encountered.^4^ We present a case of one of these uncommon anomalies, fetal anencephaly, in which POCUS led to earlier detection, less invasive management, and improved patient care.

## CASE REPORT

A 33-year-old, pregnant woman at approximately 10 weeks gestation presented with mild vaginal bleeding of a few hours duration. She denied any prior bleeding or clots and denied abdominal pain and cramping. There were no other abdominal or genitourinary symptoms. Past medical history was positive for polycystic ovarian disease and two previous spontaneous first trimester abortions.

Physical examination revealed a well-appearing female with normal abdominal and pelvic inspections. Pelvic exam revealed no blood in the vaginal vault and the cervical os was closed. Abdomen was non-tender, without rebound or guarding.

The differential diagnosis included ectopic pregnancy, spontaneous abortion, threatened abortion, inevitable abortion, septic abortion, gestational trophoblastic disease, and mechanical trauma.^5^

**Figure 1A–D:**
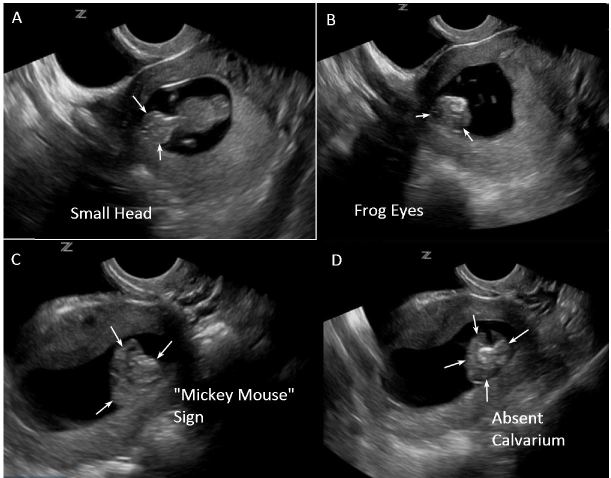
**A.** Small head. Coronal axis view of the fetus demonstrating a smaller than expected fetal head (arrows). The fetal head is noticeably smaller than the torso. **B.** Frog Eye Sign. Coronal view of the fetal head demonstrating protruding orbital structures (arrows) consistent with the “frog eye sign” and anomalous development of the fetal cerebrum as seen in acrania-anencephaly. **C.** Mickey Mouse Sign. Coronal view of the head and neck demonstrating the “mickey mouse sign.” The two abnormal hemispheres (the ears) are noted without an associated cranial vault (arrows). **D.** Small head, absent cranium. Transverse view of the fetal head demonstrating an absence of the fetal cranium (arrows) with the cortex floating in the hypoechoic amniotic fluid.

A POCUS was performed to confirm intra-uterine pregnancy and evaluate fetal viability. Initial transabdominal ultrasound revealed a single intrauterine pregnancy at approximately 10 weeks gestation. Fetal cardiac activity and movement were confirmed along with absence of hemorrhage or free fluid. Amniotic fluid volume was grossly adequate. During measurement of crown-rump length, the EP noted an abnormal appearance of the fetal head. Transvaginal views were obtained to investigate further. Transvaginal ultrasound revealed more detailed findings that suggested fetal anencephaly. The specific findings included a smaller than expected fetal head (as seen in Figure 1A), the “frog eye” sign and “Mickey Mouse” sign (as seen in Figures 1B and 1C, and Supplemental video), and absence of the fetal calvarium (as seen in Figure 1D, and Supplemental video).

**Figure 2A–C:**
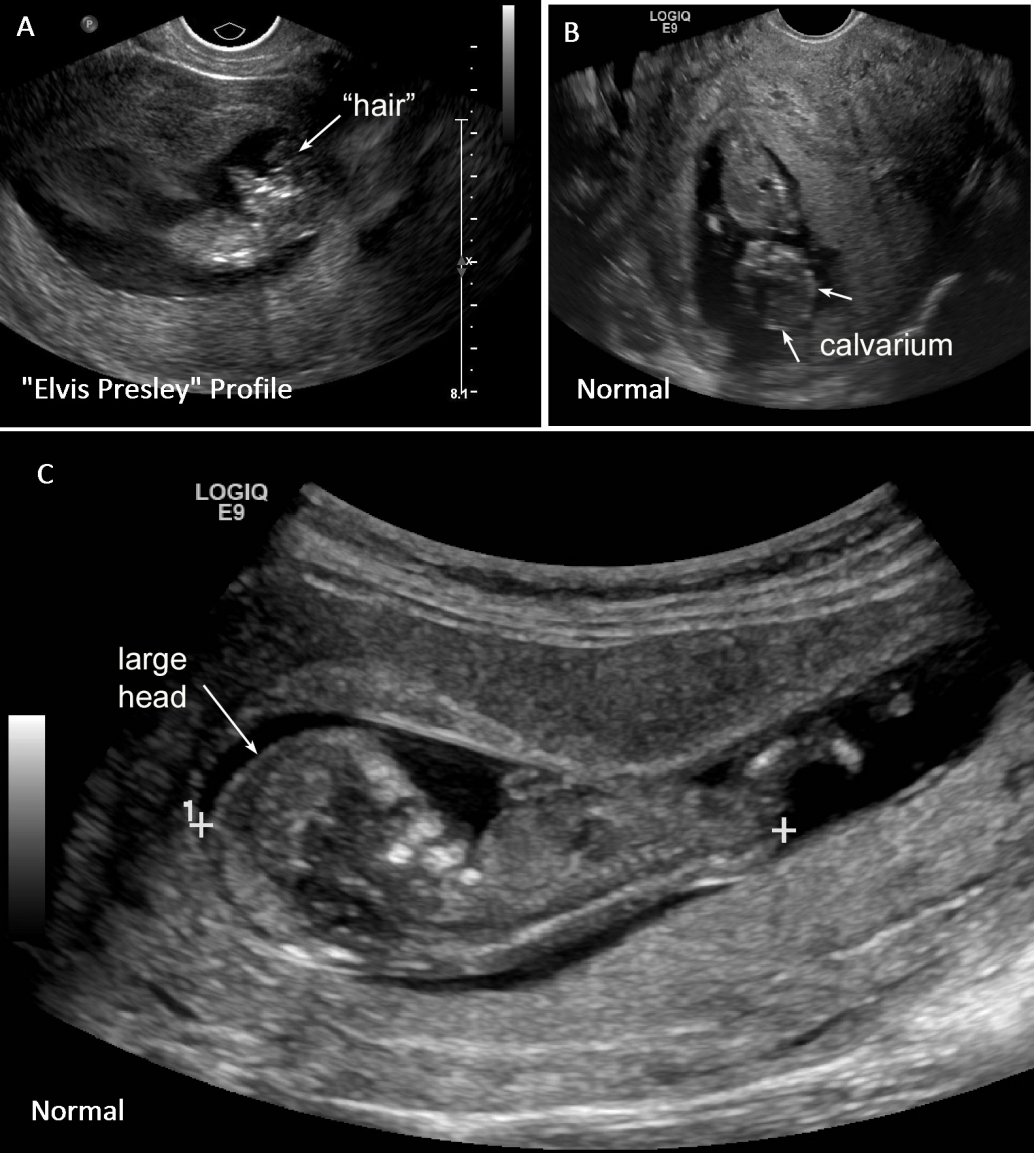
**A.** Elvis Presley Profile. Longitudinal view of the fetus demonstrating the “Elvis Presley profile”. The cerebral hemisphere is jutting forward, giving the illusion of Elvis Presley’s hair (arrow). **B.** Normal comparison. Coronal view of a normal fetus developing appropriately. Image demonstrates a normally developing cranial vault, well defined calvarium (arrows), and cerebral structures. **C.** Normal comparison. Longitudinal view of fetus developing appropriately. Image demonstrates presence of the cranial vault and a large fetal head (arrow) that is larger than the torso.

Findings also included presence of the “Elvis Presley profile” (as seen in Figure 2A and Supplemental video) (credit Debra Williams, MS, RDMS, RVT, RT(R)). Compare these abnormal findings to a normal fetus where the head appears much larger with a larger, round, better-defined fetal cranium as seen in Figures 2B and 2C (see also Supplemental video).

A short video clip detailing the common sonographic findings in anencephaly can be found in Supplemental video.

A consultative high-resolution fetal US confirmed the diagnosis, and obstetrics was consulted. The patient was counseled regarding pregnancy options. She elected for pregnancy termination and underwent a subsequent dilation and evacuation procedure. Pregnancy termination at this early gestation was lower risk; if the diagnosis had been delayed, lower-risk options would not have been available.

## DISCUSSION

In this case, an uncommon fetal anomaly, anencephaly, was discovered by the EP. Fetal anencephaly is believed to be a result of congenital lack of mesenchymal migration in the fourth week of gestation leading to absence of the calvarium and abnormal development of the cortical structures.^6^ The early recognition of this diagnosis allowed pregnancy decisions to be made earlier. The patient was able to be counseled, and lower-risk options for pregnancy termination, if desired, were available. EPs using POCUS to evaluate early pregnancy complaints should be aware of the appearance of fetal anencephaly. The sonographic findings are relatively straightforward and can be recognized during a brief routine evaluation. Early recognition of this diagnosis should improve patient care, specifically allowing earlier, lower-risk intervention if necessary.^7^,^8^,^9^

Early pregnancy complaints are common in emergency medicine and primary care. General POCUS evaluation is typically performed to confirm intra-uterine pregnancy, evaluate for signs or risks of ectopic pregnancy, and to assess fetal viability. In addition, measurements of fetal gestational age and a gross assessment of amniotic fluid volume should be carried out. During this evaluation, a brief, focused anatomic survey should be performed to confirm a grossly normal appearance of the fetal head. The normal fetal head should be relatively large, nearly the size of the torso, with a rounded cranium and the eyes centered. These characteristics can usually be appreciated from approximately 10 weeks gestation forward and can be recognized by EPs with some experience in early pregnancy ultrasound.^10^ Evaluation of the fetus can be done rapidly.^11^

In diagnosing fetal anencephaly using POCUS, a very important finding is absence of the fetal calvarium. Two diagnostic signs have been described to aid in diagnosis: the “Mickey Mouse” sign and the “frog eye” sign. The “Mickey Mouse” sign depicts the fetal cortex floating in the amniotic fluid without cranial structures above it, giving it the look of Mickey Mouse ears.^7^ The “frog eye” sign depicts the protruding orbital structures associated with anomalous development of the cortex seen in anencephaly-acrania.^12^,^13^ Other findings may include echogenic particles in the amniotic fluid consistent with fragmented cortex that occurs during the transition from acrania to anencephaly,^14^,^15^ polyhydramnios,^16^ and a crown-rump length falling below the fifth percentile.^7^

Ultrasound is the ideal imaging method for the early detection of fetal anomalies given its high diagnostic capacity, non-invasiveness, rapid detection, low cost, and availability.^8^,^17^ Moreover, ultrasound offers the advantage of earlier detection beginning at 10 weeks gestation^10^ to allow more comprehensive parent counseling and earlier decisions regarding the future of the pregnancy.^15^,^18^,^19^,^20^. Early ultrasound may help to avoid additional imaging, multiple healthcare visits, and provide EPs with a rapid method to ascertain the etiology of early pregnancy complications.

Public concern regarding fetal cranial anomalies has increased in recent months with the emerging threat of the Zika virus. The Zika virus has been shown to cause fetal cranial abnormalities, specifically microcephaly.^21^ Zika virus is being identified more frequently in the United States and may carry with it an increased incidence of fetal cranial abnormalities.^22^ The World Health Organization (WHO) declared Zika virus a Public Health Emergency of International Concern (PHEIC) in February 2016.^23^ Zika virus is an RNA flavivirus transmitted primarily through the Aedes spp. mosquito vector, although sexual transmission has also been documented.^24,25^ Officials in Brazil first noted an increase in the number of fetal microcephaly cases in late 2015, and have since demonstrated a 20-fold increase in the incidence of fetal microcephaly in areas where a known Zika virus outbreak was occurring.^23,24,25^ A similar retrospective study conducted in French Polynesia demonstrated a similar association during a Zika virus outbreak in 2013–2014.^23,24, 25^ With the presence of Zika virus in South America and ease of international travel, we can expect to see rates of Zika virus infections in the US rise in the coming years.^25^ In light of this emerging threat, it becomes increasingly important to recognize fetal cranial abnormalities early. As emergency physicians are on the front line of care for early pregnancy complaints, taking the time to quickly survey the fetal head during pregnancy POCUS can be an important step in early recognition of these abnormalities.

In summary, we report a case of anencephaly-acrania diagnosed by emergency POCUS in a pregnant female in her first trimester. To the authors’ knowledge, this diagnosis has not been reported in emergency medicine literature. Ultrasound is a rapid, cost-effective, noninvasive, accessible tool for the diagnosis of fetal abnormalities and the differentiation between specific diagnoses with regards to vaginal bleeding during the first trimester. Although the evaluation of complex fetal abnormalities is not part of routine emergency POCUS, routinely performing a brief survey to evaluate for a normal fetal head may allow early recognition of the important diagnosis of fetal anencephaly with subsequent benefits in patient care. In any unclear or concerning case, consultative ultrasound should be pursued as soon as feasible. Emergency physicians should have a low threshold for performing routine point-of-care ultrasound in pregnant women presenting with bleeding in early pregnancy.

## Supplementary Information

Video Annotated and narrated clip demonstrating the common sonographic findings associated with acrania-anencephaly including a smaller than expected fetal head, the Frog Eye Sign, the Mickey Mouse Sign, and an absent fetal cranium.
